# A Geospatial Information Grid Framework for Geological Survey

**DOI:** 10.1371/journal.pone.0145312

**Published:** 2015-12-28

**Authors:** Liang Wu, Lei Xue, Chaoling Li, Xia Lv, Zhanlong Chen, Mingqiang Guo, Zhong Xie

**Affiliations:** 1 School of Information Engineering, China University of Geosciences, Wuhan, China; 2 National Engineering Research Center for GIS, Wuhan, China; 3 Development and Research Center, China Geological Survey, Beijing, China; University of Aberystwyth, UNITED KINGDOM

## Abstract

The use of digital information in geological fields is becoming very important. Thus, informatization in geological surveys should not stagnate as a result of the level of data accumulation. The integration and sharing of distributed, multi-source, heterogeneous geological information is an open problem in geological domains. Applications and services use geological spatial data with many features, including being cross-region and cross-domain and requiring real-time updating. As a result of these features, desktop and web-based geographic information systems (GISs) experience difficulties in meeting the demand for geological spatial information. To facilitate the real-time sharing of data and services in distributed environments, a GIS platform that is open, integrative, reconfigurable, reusable and elastic would represent an indispensable tool. The purpose of this paper is to develop a geological cloud-computing platform for integrating and sharing geological information based on a cloud architecture. Thus, the geological cloud-computing platform defines geological ontology semantics; designs a standard geological information framework and a standard resource integration model; builds a peer-to-peer node management mechanism; achieves the description, organization, discovery, computing and integration of the distributed resources; and provides the distributed spatial meta service, the spatial information catalog service, the multi-mode geological data service and the spatial data interoperation service. The geological survey information cloud-computing platform has been implemented, and based on the platform, some geological data services and geological processing services were developed. Furthermore, an iron mine resource forecast and an evaluation service is introduced in this paper.

## Introduction

Following the development of information technology, the revolution in Earth information technology has continued. In the geospatial sciences, various challenges related to data intensity, computing intensity, concurrent access intensity and spatiotemporal intensity have emerged. These challenges require a computing infrastructure that better supports the discovery, accessibility, processing and utilization of data and provides a more reliable and scalable service for massive numbers of concurrent users[1,2]. The same is true in the geological domain, where research and development typically produces and analyzes large volumes of distributed heterogeneous geospatial data sets[3]. The applications and services that use geological spatial data have certain features, such as being cross-region and cross-domain and requiring real-time updating, focus on a certain application or service (e.g., the evaluation of mineral resource potential, the evaluation of geological disasters and the environment, and the evaluation of underground water)[4]. Furthermore, the geological spatial data that must be mobilized are usually distributed in different regions, different domains and different departments. Distributed geographic information processing (DGIP) has become increasingly important in the past decade with the popularization of computer networks, the growth of distributed data repositories, and the collaboration of researchers, developers, and users among multiple disciplines using geographic information[5]. To implement a collaborative, real-time functioning system, the isolated geological spatial data, services and computing resources should be logically integrated and made shareable. Information must be extracted from accumulated geological spatial data by comprehensively analyzing the data; then, the information should be transformed into knowledge that is significant for geological research[6]. DGIP plays a critical role in integrating the widely distributed geospatial resources to support the envisioned digital earth for utilizing a wide variety of information[7]. Requirements from the global initiatives and the nature of distributed geographic information call for the research and development of effective DGIP[5]. In addition, the sharing of large volumes of data sets encourages researchers and organizations to focus on consensus development of standard protocols and tools to publish and interoperate these large volumes of data sets[8]. Data semantics play an extremely important role in spatial data infrastructure by providing semantic specifications for geospatial data and in this way enabling data sharing and interoperability[9]. However, the management of resources in a distributed computing environment is inherently more difficult[10]. The use of an efficient mechanism to store, manage, retrieve and discover spatial information and services to provide the fusion and strategic decision-making ability of massive dynamic global geospatial information is significant[9,11,12]. Therefore, a GIS platform that is open, integrative, reconfigurable, reusable and elastic would represent an indispensable tool for enhancing geological information processing and services.

Cloud computing overlaps with some concepts of distributed computing and grid computing[13]. The goal of cloud computing lies in sharing of resources; however, resource sharing is not restricted to software and data; it is extensive and includes computing resources, storage resources, and knowledge resources[14,15]. With a cloud computing platform, users requisition computing power, storage, and other services gaining access to a suite of elastic IT infrastructure services as demands[16–18]. In a cloud environment, users have a large pool of easily usable and accessible virtualized resources[15,19]. Cloud infrastructure services, also known as Infrastructure as a Service (IaaS), including physical machines, networks, storage and system software, as virtualized computing resources[1,14,20]. The method of resource organization is an important aspect of a cloud computing environment. In terms of resource management or organization in a distributed environment, there are primarily three models: the resource pool model (a centralized model), the global-local resource model and the peer-to-peer (P2P) model.

Resource pooling is a mechanism for virtualizing and managing resources as a resource pool[21]. It is a centralized strategy for resource allocation and management[22]. The global-local resource model is used in Globus. It consists of four components: a resource agency, a collaboration assigner, a resource information service component and a resource arrangement manager. In the global/local-to-layer model, resource request processing is divided into local and global components[23]. Considering the availability and practicability of a universal description discovery and integration, peer-to-peer architectures have been proposed. A P2P system consists of a number of decentralized distributed network nodes that are capable of sharing resources without centralized supervision[24]. Based on the resource location, there are two main types of P2P structures: the message flooding and the distributed hash table (DHT). In message flooding, the query is propagated to all nodes in the network. However, the quantity of messages in the network rapidly increases as the number of nodes in the network increases, thereby easily resulting in saturation[24,25]. The distributed hash table method has been widely used for resource locating[26–28]. However, it is difficult to maintain the DHT when the node is modified.

Many organizations have begun to adopt cloud computing to better utilize computing resources by taking advantage of its scalability, lower costs, and easy accessibility[29]. Furthermore, various geospatial systems based on cloud computing have been developed. Some organizations have built their applications or systems on a commercial cloud that is provided by Google, Amazon or Microsoft. For example, “ModflowOnAzure” is a scientific modeling service that enables large-scale ensemble runs of groundwater flow models to be easily executed in parallel in the Windows Azure cloud[30]. Combined with the web-processing service, a geoprocessing cloud platform, AWT, that integrates Amazon cloud computing and geoprocessing functions was built to provide geoprocessing competence in a distributed web environment[16]. Various researchers have adopted a distributed computing architecture that is designed to solve a certain geospatial problem. A cloud-based framework for a spatial web portal (SWP) has been proposed to integrate several cloud features to support the SWP operation[31]. A prototype for sharing geographical analysis models that constructs a volunteer-style sharing mode for modeling and computing resources in an open environment on a cloud computing platform has been proposed[32]. The state-of-the-art application, which runs in a cloud computing environment, is composed of a wildfire risk and wildfire spread simulation service. The above two applications are delivered within a web-based interactive platform to fire management agencies as Software as a Service (SaaS)[33]. GeoSearch is a distributed search engine that leverages a series of existing standards, technologies and geospatial cyber infrastructure components to narrow the gap between users and geospatial resource providers/publishers; in addition, it hides the complexity of GCIs[6]. Grid Services for Earth Observation Image Data Processing has been proposed to cater to future Earth observation application requirements for the digital Earth. It provides the capability of addressing application requirements, such as real-time monitoring, time series data processing and processing with user-required characteristics, to meet the requirements of end user applications[34]. Geopot is a cloud-based geolocation data service for mobile applications[35].

Various cloud computing systems for geospatial information have been established; however, numerous problems must be solved, especially in the geological domain. For instance, in term of spatial data discovery and management, most current systems discover and manage spatial data using its metadata. A centralized repository of metadata with distributed data sources provides extremely fast search results to the user[36]; however, this remains a centralized way of managing the metadata in a distributed environment. In term of spatial data processing, some intermediate result data cannot be discovered by users because this type of data represents a temporary result, which cannot be registered in the system. However, this type of data is sometimes valuable. To address these problems, we designed a p2p node manager and include it in the cloud system to provide resource discovery and management, thereby making each node in the cloud system absolutely independent and distributed. In addition, the p2p node manager creates the concepts of perpetual resources and temporary data resources to manage, register and reuse the valuable intermediate results. In this paper, we propose a readily available, integrated data and service working environment that is based on a cloud computing architecture, the geological survey information cloud-computing platform (GSICCP), to share geological spatial information and geological spatial analysis models. In this environment, the geological ontology theory is imported and used to describe, organize and manage the heterogeneous geological data. Each node in the system remains independent, but the heterogeneous data, services and computing resources on different nodes were integrated and logically shared. The users assembled specified nodes using virtual organization to obtain the on-demand services. Additionally, a node management component based on a P2P pattern was adopted to organize and manage the data, services and computing resources on each node. The remaining sections of the paper are structured as follows. Section 2 proposes a cloud platform architecture for sharing geological information and services. Section 3 introduces a description, organization and management strategy for the heterogeneous geological data. In Section 4, a cloud service workflow and a P2P strategy are proposed to provide users with a transparent service environment. Section 5 introduces some the related performance test results and use cases of geological cloud platform. The prospects for the geological cloud platform are discussed in Section 6.

## Hierarchical Architecture of the GSICCP

**Fig 1:**
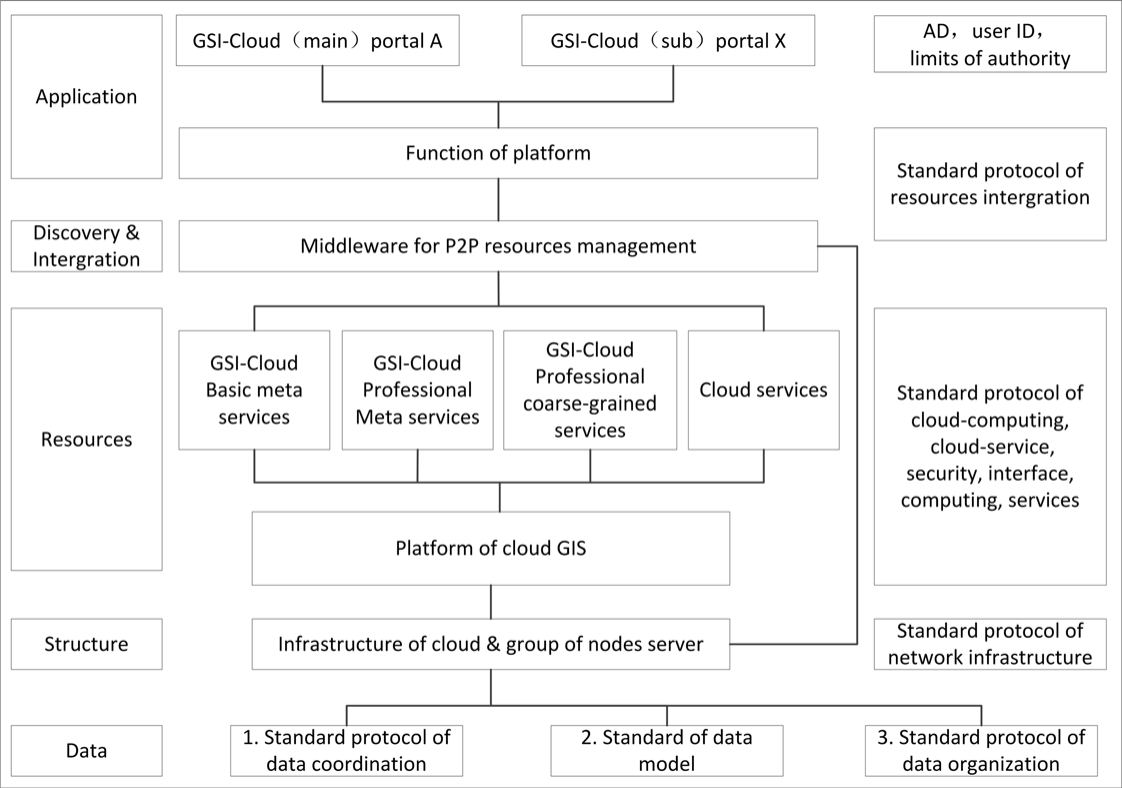
Hierarchical architecture of the geological survey information cloud-computing platform.

Using the Chinese geological ontology description, adopting the P2P architecture, taking the Geographic Information System as the principal source, focusing on resource agglomeration, and employing a cloud concept strategy, we designed a distributed geological survey information cloud-computing platform and organized the hardware, software and data resources by building a resource aggregation standard and protocol on this platform (Fig 1).

According to Fig 1, the GSICCP is divided into the following layers: data, fabric, resources, discovery and integration, application and representation in the vertical direction. The cloud platform architecture is built on multiple compulsive standards that attempt to eliminate an existing resource islet and prevent a new resource islet from appearing. The following are the contents of each layer:

1. Data layer: This layer consists of multi-level standard-based databases and is responsible for the integration of multi-source, multi-scale and multi-temporal geological data, including graphical data or data that is organized according to non-standard database models, databases built using multi-period data models, databases organized according to a large databases, or thematic databases organized according to a data warehouse technology. In this layer, we adopt a geological domain ontology theory as the foundation for unified organization, semantics sharing, discovery and integration to build a universal description and organization model for the geological spatial feature data in a distributed environment and to provide a framework for geological data service inter-operation.
2. Fabric layer: Grid nodes form the foundation of the GSICCP. This layer connects the infrastructure and node server groups of each professional unit and integrates the hardware and software resources according to the network. The hardware resources primarily include multi-level servers, personal computers, mobile phones and location terminals. The software resources primarily include the operating systems, such as Windows, Linux, Unix, and Aix; the development frameworks, such as.Net; and the GIS platforms such as MapGIS, ArcGIS, and SuperMap. The GSICCP combines a dedicated network and the Internet and employs various security strategies, such as a software firewall, a hardware firewall and other internet security technologies, to build the net system.
3. Resources layer: This layer is the foundation of the GSICCP. It consists of pivotal modules, including the GSI-CLOUD resource integration middleware, the GSI-CLOUD meta service libraries for different granularities and functions, the GSI-CLOUD workflow service middleware, and the GSI-CLOUD service engine middleware.
4. Discovery and integration layer: This layer is the core module of the GSICCP. The purpose of this layer is to provide a node management method that combines the distributed resources in the cloud environment and universally describe the hardware, software and data resources. In this layer, P2P node management middleware is adopted to implement resource organization and management. This middleware primarily includes a virtual node resource integrator, a cloud node meta service library, a virtual node portal configurator and a temp data resource integration container.
5. Application and representation layer: this layer consists of a geological survey information cloud portal and relevant coarse-granularity service modules. The GSICCP achieves the basic functions and provides the basic architecture and public cloud in terms of resource sharing and cooperation (as a public cloud). In this layer, different requirements and professional applications can be deployed on the platform in the form of a service. The users only need to pay attention to their own business process and functions (as a private cloud) without paying attention to how the resources share and cooperate.

## Organization and Management of the Geological Data

### Geological Ontology

Building a geological data model is the foundation for multi-source, multi-scale, heterogeneous geological spatial data integrated organization and management. The model describes the data content, structure, behavior and semantics to assist in forming a common comprehension. The existing data models or modeling methods emphasize the project requirements and are accompanied by personal database technology limitations. Therefore, this suggests that geological modeling lacks a consistent description of the data and data relationship, the semantic restraint rules related to the formal description, and the data content, structure, behavior and semantics that are governed by the data model. Thus, the abovementioned problems influence the shared understanding of geological scientific data and limit the ability to design a basic data model using various data structures.

The ontology is concerned with a conceptual structure of the methods that we use to describe the world[37,38]. It is an explicit specification of a sharing conceptualization, which is used in an integration task to describe the semantics of information sources and to make the contents explicit. Additionally, it is used to identify and associate the semantically corresponding information concepts[39]. In recent years, the ontology concept has been used in the information domain to solve problems related to knowledge concept expression, knowledge organization structure, knowledge sharing and semantic consistency. These problems play a pivotal role in supporting information reuse, sharing and exchange. Therefore, geological ontology research is important. Geological ontology provides a foundation for geological professional and application standards. Therefore, it is the guiding model for the entire geological data model. Additionally, it is the basis for integrated geological data description, organization and discovery and for achieving inter-operation between different systems, geological ontology supplies, geological knowledge ontology and knowledge mining services for professionals and non-professionals.

Geological ontology research builds the description of objects and relationship between objects in geological domain. The framework of the geological ontology consists of a domain outline table, a basis category table, a main table (a detail table) and a redistribution table (a typical class). The domain outline table consists of the primary categories of the geological ontology classification, and it defines the basis subject category and the arranging sequence. The basis category table consists of the basis broad heading and the second and the third categories. Therefore, it is the classification category framework that helps users understand the general classification situation. The main table is a list that contains various categories. It is the principal part of the ontology, and it is the basis of the classification index. The redistribution table uses the existing common geology categories as a typical class to describe and adopt the assembled technology. The redistribution table uses a simple category number that expresses a simple theme concept, constructs a composite category number according to certain rules, and expresses a complicated concept, which is in the classified table.

### Geological Spatial Data Organization and Management

Using the geological ontology theory and the spatial data concept model, geological map data and tile pyramid data models were adopted to organize the geological spatial data in the GSICCP; furthermore, the MapGIS platform was employed to manage the geological data. The MapGIS platform adopts a service-oriented architecture (SOA) and a multi-level structure, and determines the spatial content and its relationship-oriented data organization. Furthermore, it facilitates the effective storage and indexing of the massive spatial data. Therefore, the MapGIS platform supports a distributed spatial data calculation in local and wide area networking environments. To integrate the heterogeneous data from other GIS platforms, such as ArcGIS, SuperMap and GeoStar, the middleware technology was employed to help share the multi-source data on the GSICCP.

**Fig 2:**
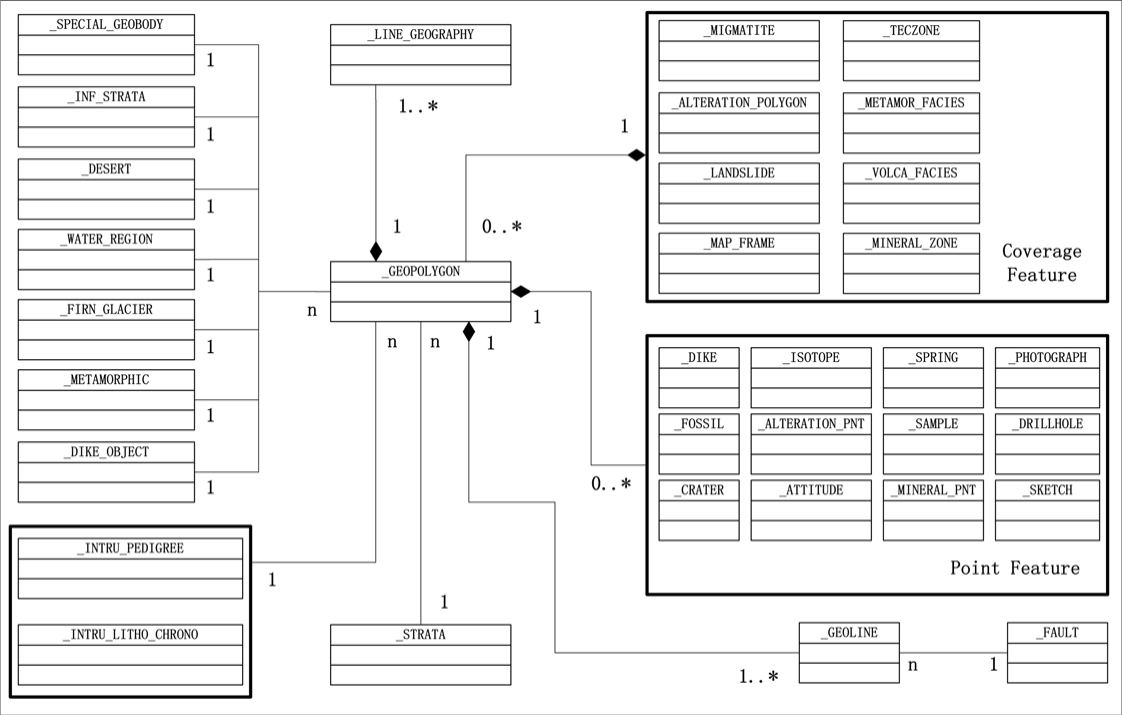
Data model framework of the geologic map.

The geologic logical data model was built according to the geological survey requirements and an existing data modeling method using the geographic information application model rules (ISO 19109), the geographic spatial application model (ISO 19107), the feature description framework, UML, and the relational database normalization theory. Fig 2 shows the geological spatial data model framework. This framework is a specialized standard for the geological map data model that uses the specialized standard application model rules; additionally, it can be transformed into a multi-scale geological map data model.

**Fig 3:**
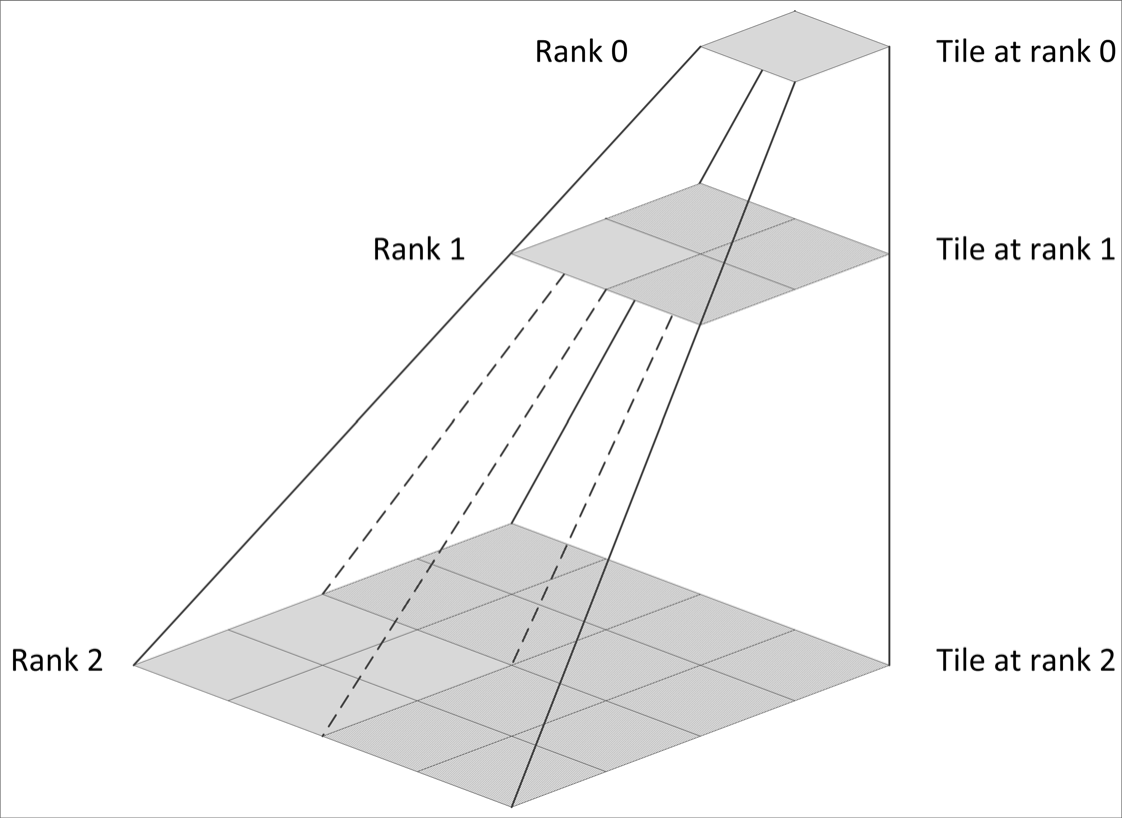
Tile pyramid data model.

In the grid stream geological map data service, the tile pyramid model was adopted to organize the multi-scale geological map. As shown in Fig 3, the top pyramid layer in rank 0 shows the original geological map panorama. The tiles in rank 1 are divided according to the rank 0 tile in the 2×2 form and used to generate four tiles in a sequence. The tiles in rank n are divided according to the rank n-1 tile in the 2×2 form. Thus, it is easy to calculate the number of tiles in the L rank according to the following expression:

$$n=\sum_{i=0}^{L-1}2^{i}\times 2^{i}\;(L>0)$$ (1)

**Fig 4:**
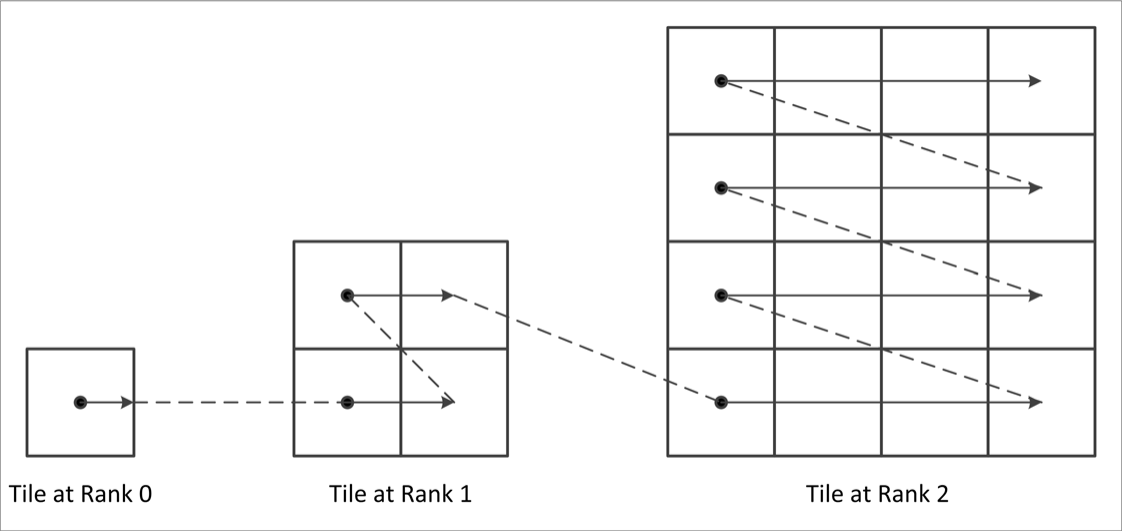
Numbering method for each tile.

**Fig 5:**
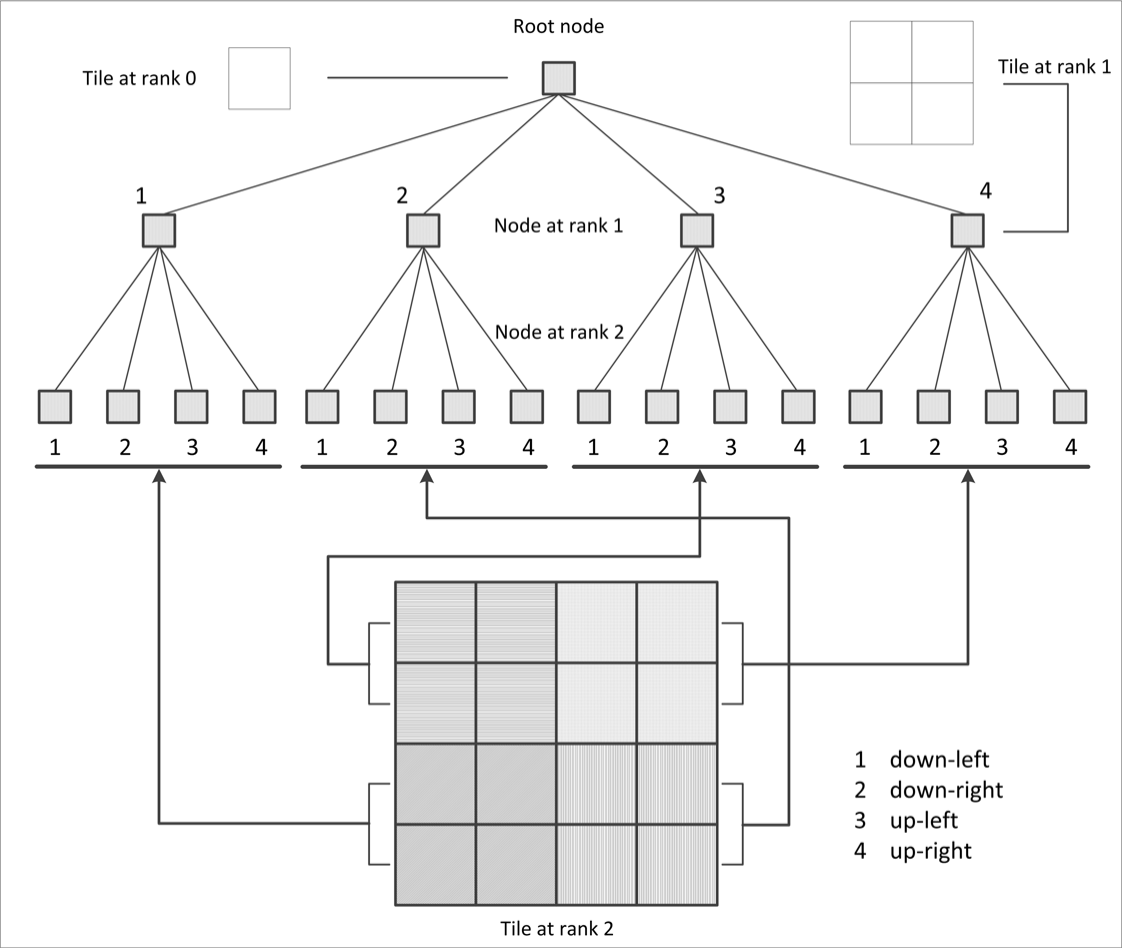
Corresponding relationship between the tile number and the quadtree node.

As shown in Fig 4, the serial number of each tile (from the highest layer at rank 0 to the lowest layer at rank n, from bottom to top in the same layer and from left to right in the same row) increases by a degree, and each serial number corresponds to the table number displayed to the client. The indexing for each tile is completed using a quadtree. The quadtree traverses each node in a top-down approach (rank-by-rank). In each rank, the quadtree traverses the node from left to right. Thus, as shown in Fig 5, the first, second, third and fourth node from left to right in each rank corresponds to the bottom left, bottom right, top left, and top right tile in the pyramid, respectively.

## Workflow and P2P Node Management Mechanism

### Hybrid Framework of the GSICCP

**Fig 6:**
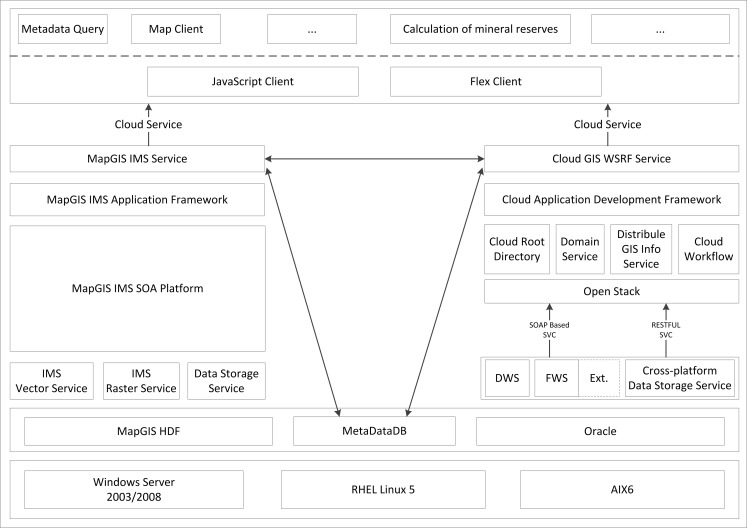
Hybrid framework of the geological survey information cloud-computing platform.

The GSICCP is based on the cloud GIS platform, which completes the platform architecture by combining a P2P node management mechanism with the cloud GIS middleware. The cloud application development framework publishes functions to users in the Cloud Service forms and thus provides a customized rapid development process for users. The GSICCP develops a hybrid framework (Fig 6) that integrates the IMS and Cloud GIS WRSF Services based on the WSRF. Furthermore, it provides the GSICCP with an ability to be deployed and run on heterogeneous operating systems such as Windows, Linux and Unix. Additionally, it supports different data formats such as local spatial data formats (e.g., MapGIS HDF) and spatial database formats based on large commercial databases (e.g., Oracle 10g/11g, IBM DB2). In the foundation of the cross-platform GIS C/C++ kernel, the GSICCP packages the low-level GIS functions by adopting JNI technology. Then, the GSICCP publishes the low-level GIS functions as a meta function service in the forms of SOAP and REST and provides a traditional service API and a non-status service API to a higher level of the framework. The open stack is used to package the API, which was provided by the meta function service layer, and generates a series of cloud GIS function components such as the cloud root directory and domain object management, distributed spatial calculating, cloud workflow and user security management components. Based on these cloud function components, the Cloud Application Development Framework is formed. All of the underlying functions are published to the upper layer by the cloud service, thereby allowing users to rapidly customize and develop their own applications in the portal layer using rich client development technologies such as JavaScript and Flex.

The GSICCP adopts the domain-based business integrated approach, retrieves the eligible resource nodes according to the root directory manager component, builds the dynamic virtual organization in the cloud environment, saves the domain-related information (e.g., domain manager node ID, domain ID, domain node information description, and domain service description), creates the domain resource directory and registers the domain objects on the global domain directory management node. When domain objects are modified, the manager and the global directory service nodes are joined to determine the modified information. If a node in this virtual organization fails to function, the global domain management service generates a new domain management node for this virtual organization.

This type of hybrid integrated architecture maintains the original system stability and intentionally imports the cloud GIS function component, which improves the efficiency and maintainability of each node.

### Cloud Service Workflow

In the GSICCP, the Geospatial Cloud Service Workflow System is adopted to promote geospatial information processing from a desktop to a cloud environment. According to the Geospatial Cloud Service Workflow System, various spatial and non-spatial information services are assembled on demand. Thus, the blending of traditional and cloud GIS services is achieved. Using the geospatial cloud service workflow management component, the users conveniently choose multiple geospatial information resources. This provides an important technological guarantee for integrating and sharing geospatial information.

**Fig 7:**
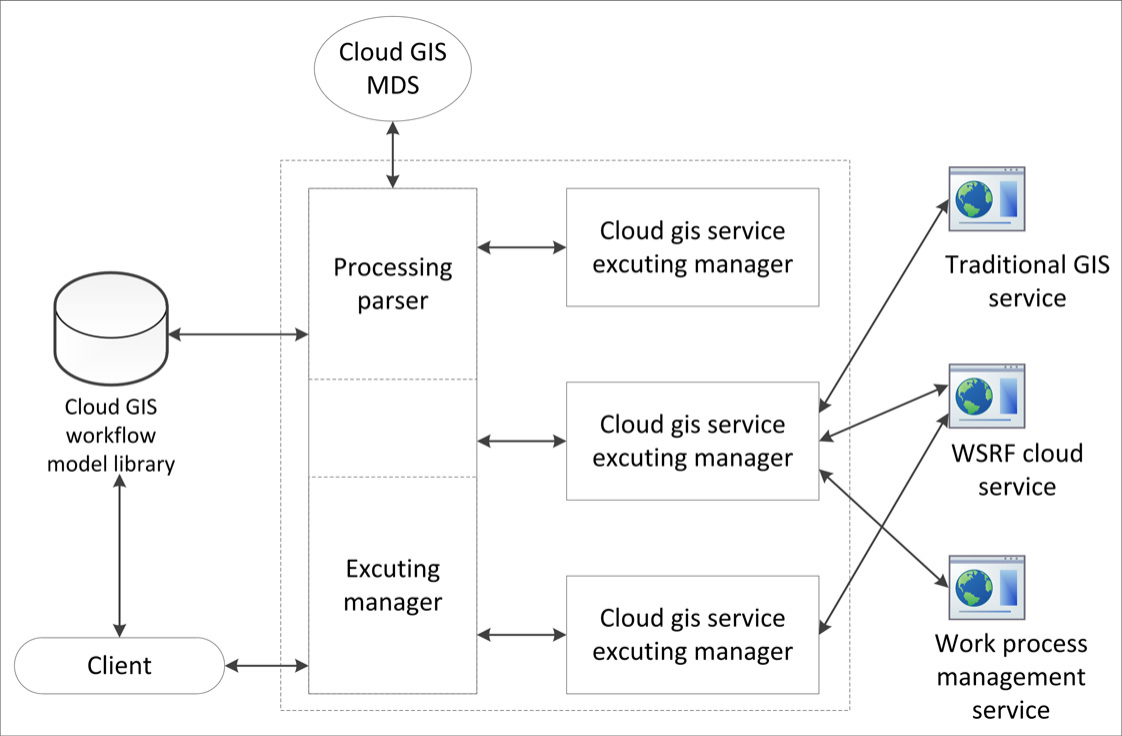
Distributed workflow engine deployment architecture.

The geospatial information cloud service workflow engine (GICSWE) is the core of the cloud GIS business process management. The GICSWE, according to the business description file parser and workflow execution monitor, controls and manages the GIS service flow data, execution status, flow execution result and the GICSWE architecture, as shown in Fig 7. Considering features of business flow, the global parser and a task processing manager of the GIS workflow were deployed on the same node. The task executer was deployed on another work node to form a distributed architecture. In the client, the users build a model by choosing the proper business flow units from the workflow model library. Then, these units form and generate a service flow description file. The description file will be submitted to a workflow execution manager that has a light load on one cloud node to parse the file globally and to distribute tasks to several nodes. The workflow execution manager will interact with a distributed cloud GIS service executer in real time. The P2P messaging mechanism is utilized to synchronize the execution statuses and results of the workflow between the workflow execution manager and the distributed cloud service executer.

### P2P Node Manager Mechanism

**Fig 8:**
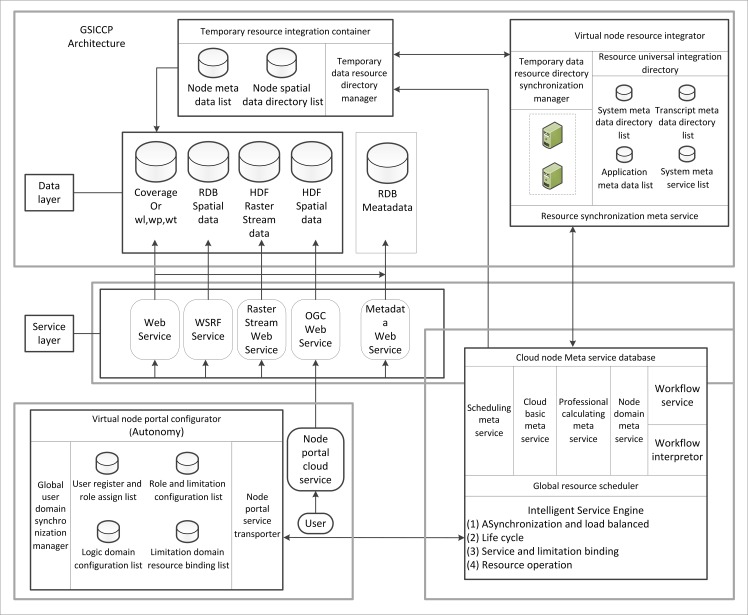
Architecture of the P2P node manager.

The P2P node manager (Fig 8) is the middleware of the GSICCP. This middleware is required to provide a service environment that maintains the autonomy of each node without changing the original environment (e.g., operating system, network protocol, or network service). Additionally, the P2P node manager ensures the users’ and nodes’ security. The lower level data information, hardware information and software information will be shielded by the P2P node manager. Thus, the users will be in fully transparent computing or servicing environments.

In the GSICCP architecture, a node does not represent different servers. The node is a group that consists of servers and personal computers. The nodes are distributed over the entire environment, and they can be physically or logically divided. To provide the features of geological spatial information applications and services, the P2P node manager adopts a virtual node resource integrator, a cloud node meta service library, a virtual node portal configurator, a temporary data resource integration container and an SOA-based resource integration model standard. Additionally, the P2P node manager provides the description, organization, discovery, integration, sharing and cooperation for distributed resources. In the GSICCP environment, according to the P2P node manager, the resource statuses are described and monitored, and all services and resources are shared; additionally, resource efficiency and service quality are improved. On a virtual node, a coarse-granularity meta service is not divided into several fine-granularity meta services. Rather, it is arranged on a suitable machine to run in-line with a loading condition.

The P2P node manager is one of the core modules in the GSICCP. Its purpose is to organize the distributed resources in the cloud and to universally describe the hardware, software and data resources. The node structure is not of a centralized form. By considering the autonomy, sharing and coordination of each node, the environment resources are divided into regional and global resources. The regional resource is managed by each node. The global resource is shared and managed by all cloud platform resources that require synchronization mechanism support. Additionally, the P2P node manager supports the long-lived transaction during the cloud computing process and cloud serving. The function of each component of the P2P node manager is described in the following:

1. Virtual node resource integrator: This integrator is used to describe, organize and manage the perpetual resources in the cloud environment and, according to the synchronism, synchronously update the global resources on each node. The virtual node resource integrator includes a system metadata directory list, an application metadata list, a transcript metadata directory, a system meta service list and a universal resource directory synchronism manager.
2. Temporary data resource integration container: This container is used to manage the new data resources generated by the service layer. The new data are an intermediate result that is generated during processing. The data will be transformed into perpetual data, which are managed by the virtual node resource integrator according to the synchronization. The temporary data resource integration container consists of a node metadata list, a node spatial data directory list and a temporary data resource directory manager. The contents of each list are the same as in the virtual node resource integrator.
3. Cloud node meta service library: This library is used to build a service layer, manage a service and assign an online hardware resource. The meta service library on each node includes 1) a meta service and a meta-computing library, 2) a workflow interpreter, 3) a global resource scheduler, and 4) an intelligent service engine.
4. Virtual node portal configurator: This configurator is used to provide a service entrance for users by connecting the users and service layer. The virtual node portal configurator consists of a user register and role assigning list, a role and limitation configuration list, a logical domain configuration list, a limitation and resource binding list, a global user domain synchronization manager and a node portal service transporter.

### Distributed Node Portal

The distributed node portal is an exemplification of the P2P pattern and facilitates resource collaboration and sharing between local and other remote nodes. Resource publishing on a local node demonstrates the autonomy of each node. The sharing and collaboration of resources are implemented by sharing the global resources and synchronization of the platform function resources. Each node in the GSICCP belongs to an independent application domain, which includes a main portal for a local node; in addition, the node visits the sub-portal on another node according to this main portal. The distributed node portals follow a decentralized model.

**Fig 9:**
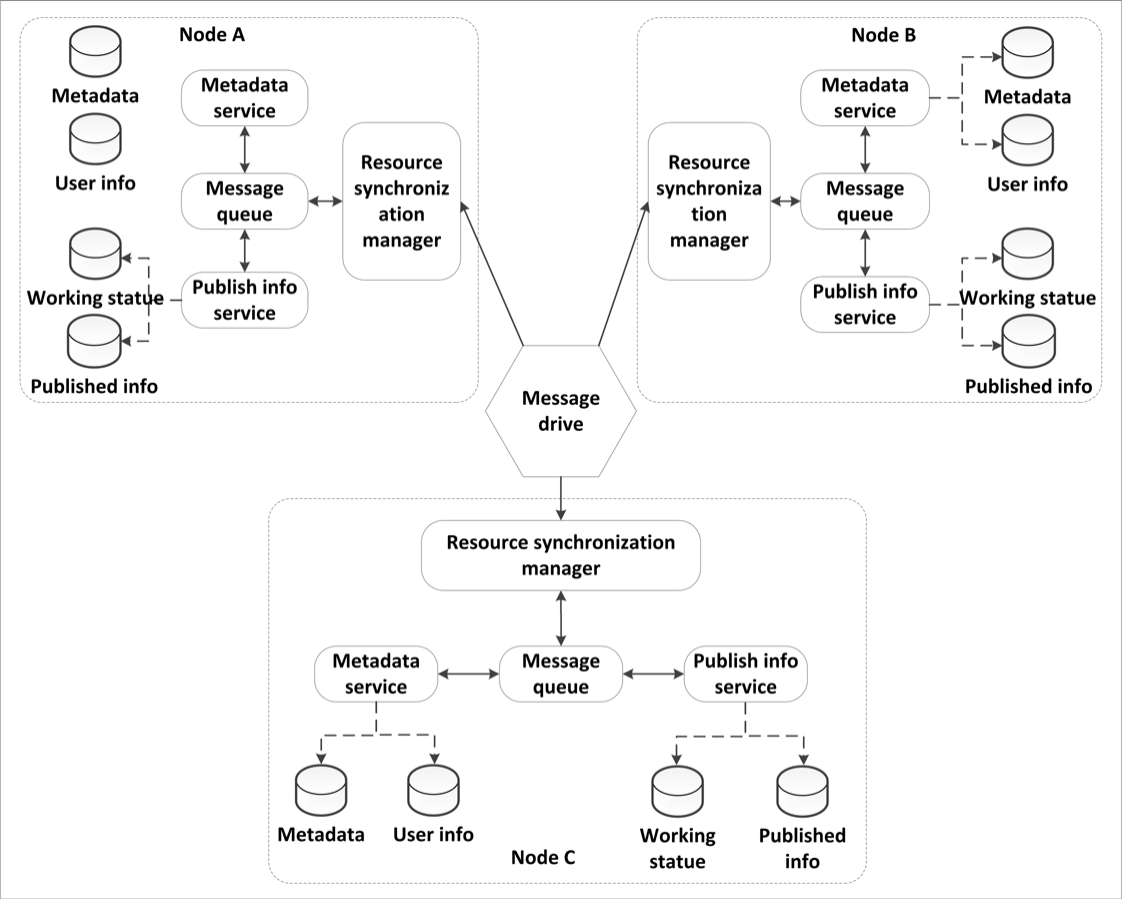
Resource synchronism of the node portal.

In the node synchronization structure, the node portal only needs to manage the resources on a local node. After finishing the registering of resources, logging out or updating locally, the related message will be pushed into a local message queue. The node portal synchronization manager will receive the registering, logging out or updating message from the message records and will then send the resource information to other nodes according to a related web service. The portal synchronization manager adopts the message mechanism to provide resource registering, logging out, updating, publishing and discovery between different nodes. Each node maintains a message queue. This message queue is different from a traditional message queue such as MSMQ (Microsoft Message Queue). In the MSMQ, the sender packages the content into a container to form a message and then places the message into a public message queue. Then, the node on a local or on a remote node receives the message that is sent to it from the public message queue and processes the message. In the GSICCP, the message queue operation is restricted on a local node; a message does not need to be sent to other nodes. The resource registering, logging out, updating, publishing and discovery operations are completed by invoking related web services on the other nodes. Fig 9 shows the node portal resource synchronism. The resource synchronization is performed between the nodes. For example, there are several nodes in the GSICCP. If new resources are registered on one of the nodes in the GSICCP, the node portal synchronization manager will invoke the metadata service or publish a service on a local node to obtain the content of the resource. Then, this node will invoke the related updating web services that are deployed on the remote node to accomplish the metadata upload.

**Fig 10:**
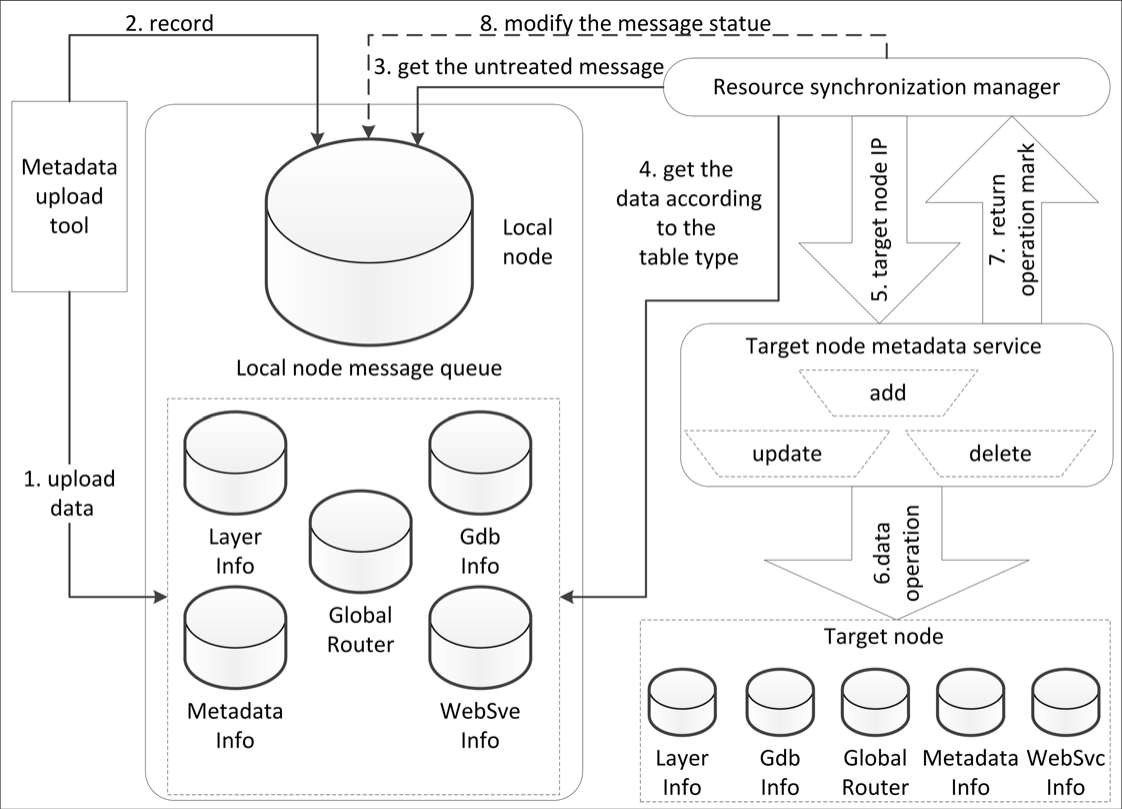
Resource synchronization process.

Each node operates and manages the data by utilizing data management tools. According to the design, each node in the GSICCP deploys the same metadata service, synchronization tools, meta database and node portal flow resource synchronism, as shown in Fig 10. The portal is one of the most important components of the GSICCP. The portal usually integrates security managing, authorization managing, node resources monitoring, and charging functions. A portal in the GSICCP is different from a routine portal because in the GSICCP, each node portal is P2P instead of being centralized.

## Experiments

### Use Case

#### Multi-mode Geological Data Service

**Fig 11:**
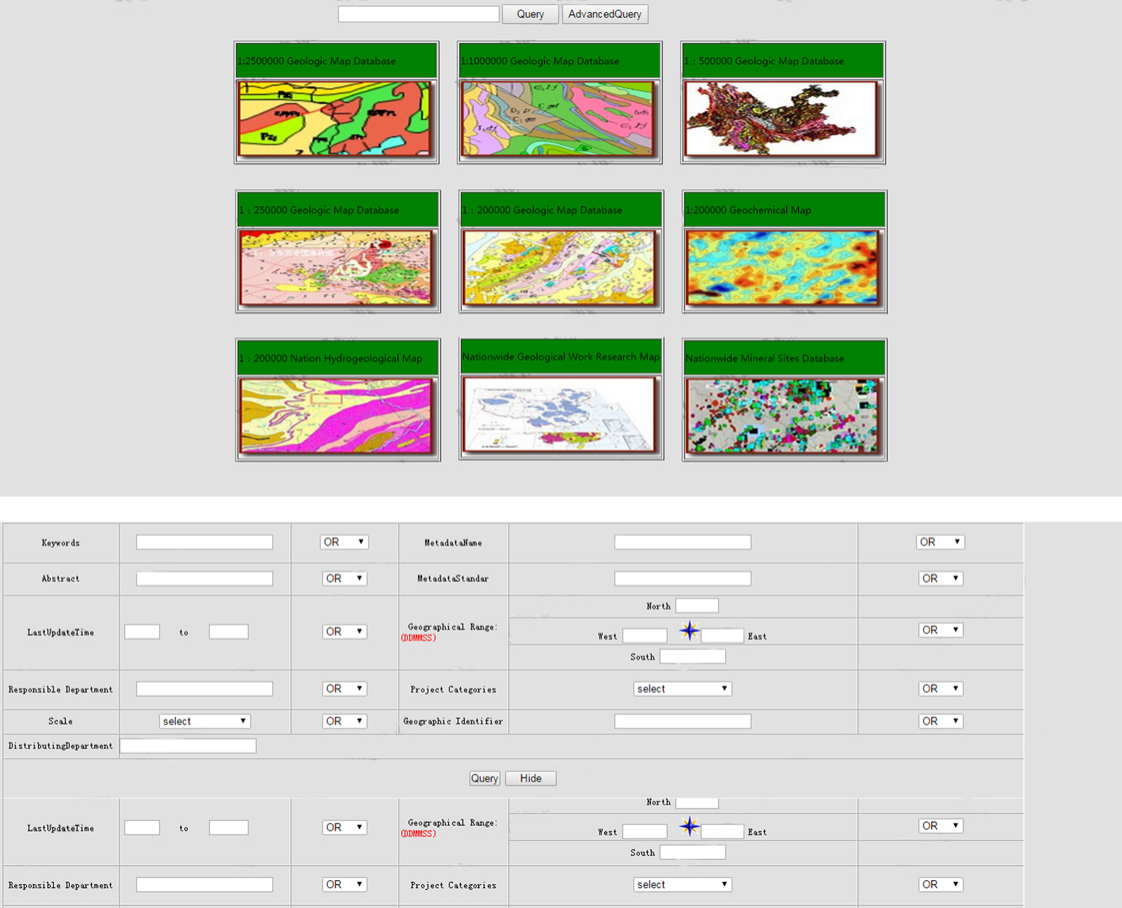
Multiple methods for metadata retrieval.

**Fig 12:**
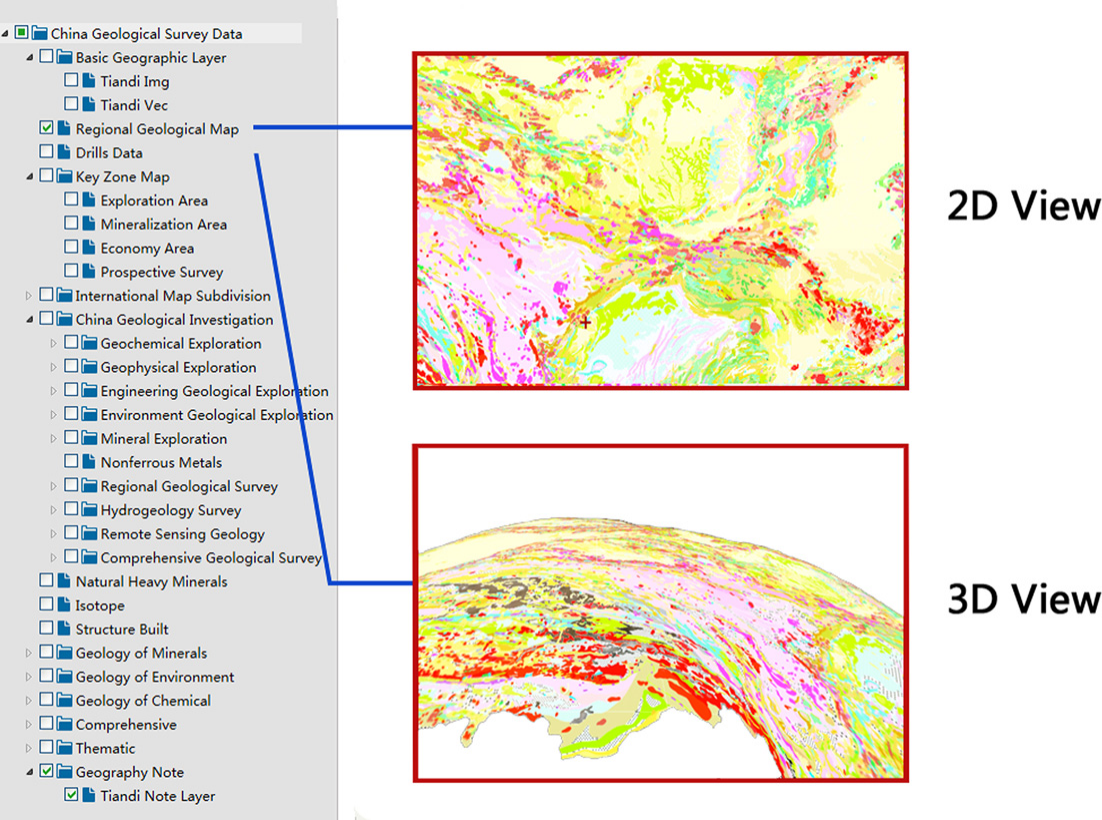
Distributed multi-source, multi-scale geological grid stream service.

**Fig 13:**
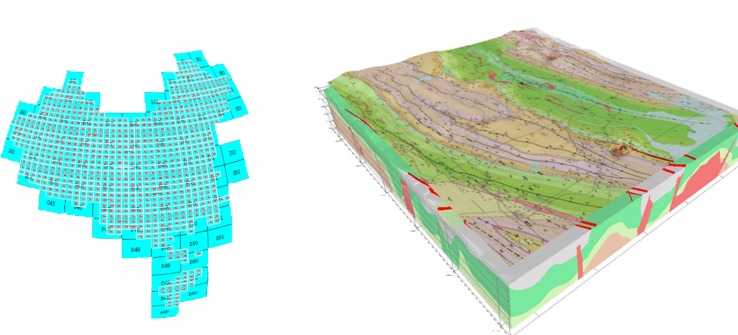
Geological integrated map service.

Based on the GSICCP architecture, the nationwide geological data are deployed on different nodes. The computing, data and software resources are virtualized into a logical integrated domain that caters to different user groups. The platform provides multi-level services (http://www.gsigrid.cgs.gov.cn/) such as the geological metadata service; the geological grid stream service; the multi-node, multi-source geological data integrated service; and the geological data visualization in 3D service. Users request these services according to the web service without knowing the data source and data distribution. Then, the users obtain a seamlessly integrated view on a web browser. Some of the most important use cases are discussed below:

(1) Geological metadata service: This service provides the data resource discovery service based on metadata. The metadata system discovery mechanism primarily seeks out the servers that store the data by retrieving the information, querying the metadata and metadata catalog tables, and finally processing the queried data (Fig 11).

(2) Geological grid stream service: This service facilitates the transformation, organization and publication of a multi-scale distributed geological map in an image format. A user obtains a service that provides information ranging from a coarse to fine granularity (Fig 12).

(3) Multi-node, multi-source geological data integrated service: This service facilitates the discovery, retrieval and integration of thematic geological data from different nodes and sources. This service includes three types of retrieval methods: a spatial extent-based retrieval, metadata keyword-based retrieval, and web service-based retrieval method. The advantage of this service is that it integrates various types of information in one view. After selecting a target area, the data catalog related to the target area is retrieved from the meta database, and the data is presented in a vector map form (Fig 13).

(4) Geological data visualization in 3D service: Using a 3D graphical interface, the service displays the geological data in a 3D form to users. The geological data are spread on a virtual 3D Earth model. When the display rank changes, the rank of the 3D image changes as well, and the users can rotate the 3D model to browse the geological data anywhere (Fig 12).

#### Geological Processing Service

The geological information processing service is complicated because it needs to establish the service flow based on a meta service. This service includes the definition, generation, storage, organization, execution management, and achievement of a spatial event and the management of a spatial information processing workflow. The meta services are assembled and invoked arbitrarily to form an execution flow and thus complete the spatial application task. According to the workflow and various middleware technologies, the service flow or a workflow that focuses on a different application or the thematic processing tasks can be packaged into the GSICCP to a certain problems or meet various requirements. The processing work is distributed on different nodes. When each node completes its work, the result of each node is compiled onto the starting node, which returns the final result to the user. Here, we introduce an iron mine resource potential evaluation service flow that is based on the GSICCP.

**Fig 14:**
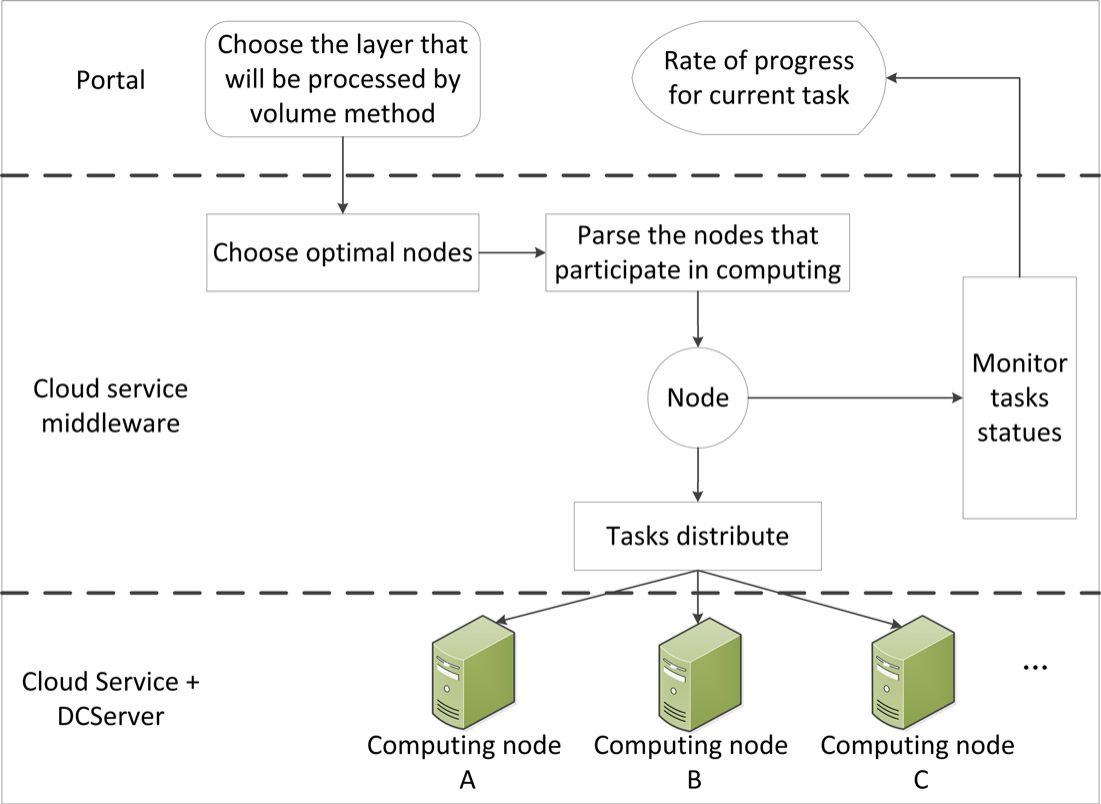
Process of GIS cloud-computing service for iron mine potential evaluation.

**Fig 15:**
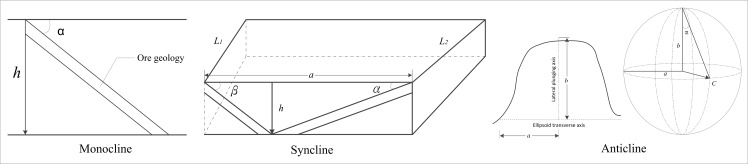
Graphical representation of the volume calculating parameter.

Mineral exploration aims to discover new mineral deposits in a region of interest[40], wherein mineral potential evaluation plays an important role, the results of mineral potential evaluations represent a significant reference for mineral exploration[4]. When using a traditional approach, the iron mine resource potential evaluation work is difficult because the geological data are distributed across various areas and departments, and multi-type geological data need to be collected, storing and analyzing the data also present a challenge[4,41]. Geo-spatial information technology is widely used in the mining industry, and it is beginning to move from traditional experience-based methods to quantitative analysis and automatic direction science-based methods[42]. However, there are limitations to evaluating the mineral potential only with the use of desktop or web-based geospatial information technology. For instance, one could not solve problems such as those resulting from incomplete information, data integration, information extraction and thematic mapping[43,44]. Distributed computing can be applied to help solve these problems[41]. Using the GSICCP, the iron mine resource potential evaluation task is easily accomplished. A workflow is established on the GSICCP to build the iron mine resource potential evaluation process. The data storage and computing are distributed across multiple nodes. Therefore, it is easy to process the vast geospatial data. The cloud GIS computing service process for the iron mine potential evaluation is shown in Fig 14. To address different seam forms, the volume calculating model can vary. There are three types of seam forms: monocline, syncline, and anticline. The volume calculating model and the graphical representation parameter for each form type are shown in Fig 15. In the following expressions, *h* represents the lower limit for prediction, *M* represents the ore geology thickness, and *L* represents the geological strike length. For monocline, the model for calculating the volume is

$$V=\frac{h}{\text{sin}\;\alpha }\times M\times L$$ (2)

For syncline, the model for calculating the volume is

$$V=(\frac{h}{\text{sin}\;\beta }\times L_{1}+\frac{h}{\text{sin}\;\alpha }\times L_{2})\times M$$ (3)

For anticline, the model for calculating the volume is

$$V=V_{2}-V_{1}$$ (4)

$$V_{1}=\frac{4}{3}\pi \times a\times b^{2}\times \text{tan}\;\alpha$$ (5)

$$V_{2}=\frac{4}{3}\pi \times (a+M)\times (b+M)\times (b\;\text{tan}\;\alpha +M)$$ (6)

**Fig 16:**
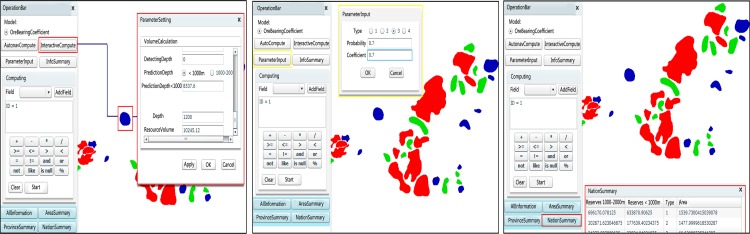
Iron mine potential evaluation on the GSICCP.

**Fig 17:**
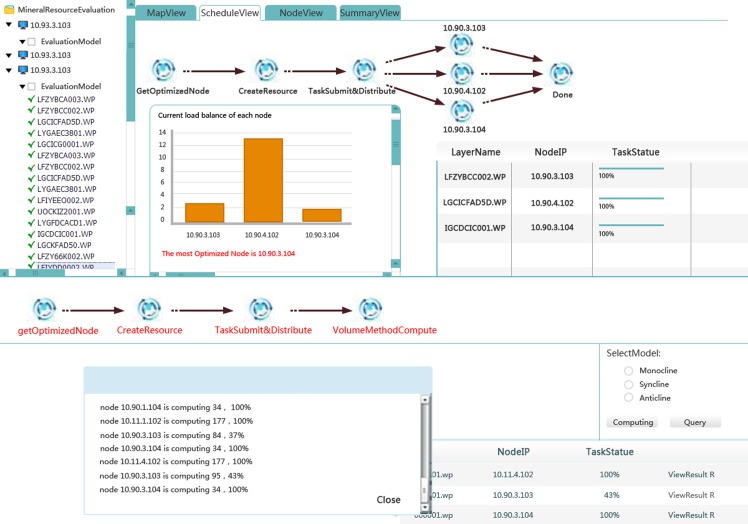
Iron mine potential evaluation workflow.

In this example, the data are multi-type, and the data volume is greater than 800 G, as illustrated by Table 1. The actual iron mine resource potential evaluation study, which uses the GSICCP, is shown in Fig 16. The workflow (Fig 17) of the iron mine resource potential evaluation is as follows: 1) choosing and integrating the multi-node data, 2) inputting the basis parameters, 3) calculating the predicted ore volume, 4) finding the target area using the evidence weight method, 5) calculating the volume, 6) summarizing the province data, 7) summarizing the area, 8) summarizing the nationwide data, and 9) outputting the results. After the evaluation process, the workflow is saved for reuse by the users or published as a service for the users.

**Table 1:** Geological data used in the iron mine resource potential evaluation.

Num	Thematic Map	Classification of Thematic Map	Quantity	Amount
**1**	Thematic atlas for Metallogenic prediction	prediction result map	135	170
		upgrade of iron ore	35	170
**2**	Thematic atlas for metallogenic regularity	typical deposits of metallogenic elements	109	387
		typical deposit prediction elements	85	387
		workbench metallogenic elements	103	387
		workbench prediction elements	70	387
		provincial map	20	387
**3**	Thematic atlas for geological background	deeds material figure	76	160
		building structure diagram	39	160
		the predicted workspace metamorphic structure for construction	16	160
		the predicted workspace intrusion magma working map	14	160
		the predicted workspace deposit structure for construction	9	160
		the predicted workspace structure lithofacies paleogeographic map	4	160
**Total**	899 metadata			717

### Performance Test

#### Map Service Performance

Requests for a geologic vector map and tile map for Wuhan, China, were used to test the performance of the map service in GSICCP. During the performance test, the entire environment was deployed as follows: 1) Hardware environment: Includes an application server, database server and GIS server, where each server has 32 GB of RAM and 8 3.86 GHz CPUs, connected by a 1000 Mb network. However, due to certain limitations, each server only uses 1 CPU to participate in this test. 2) Software environment: The GSICCP portal was deployed on each application server, the database server utilized the Oracle database to store the spatial data, and MapGIS IGServer (a MapGIS software package for map service publication) was deployed on the GIS server to provide the related spatial information service.

**Fig 18:**
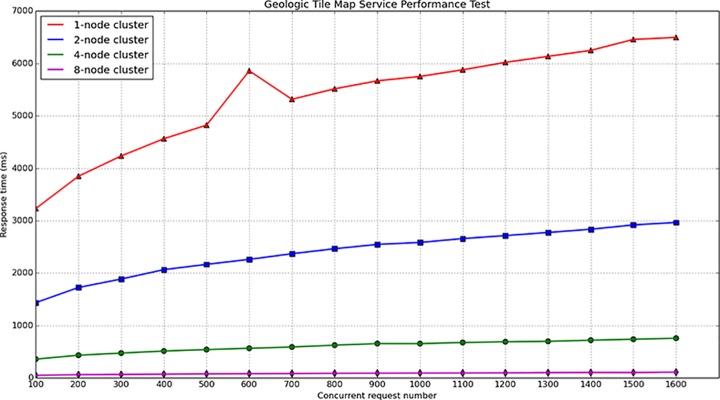
The geologic tile map service performance test results.

**Fig 19:**
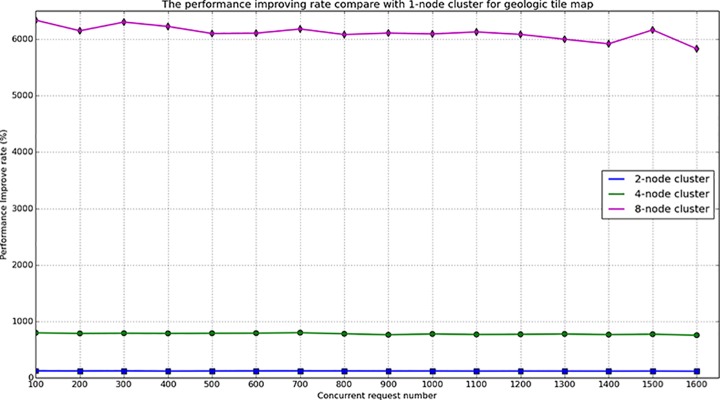
Performance improving rate of tile map service compared with 1-node cluster.

**Fig 20:**
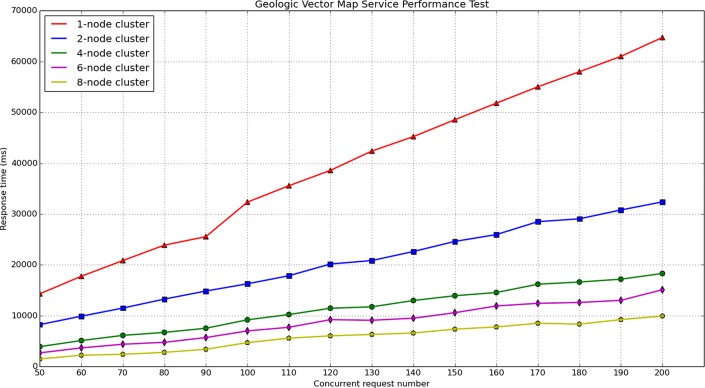
The geologic vector map service performance test results.

**Fig 21:**
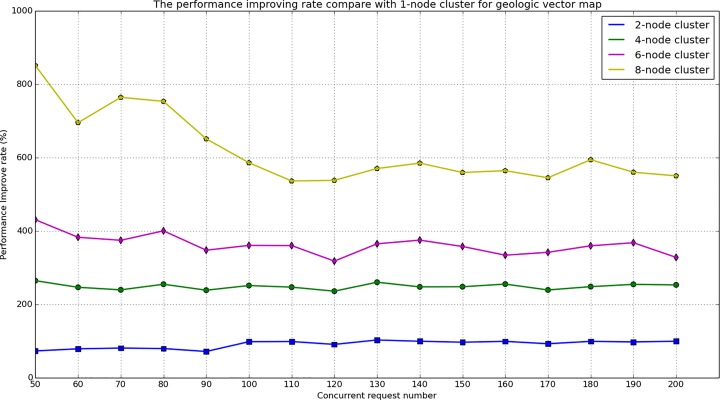
Performance improving rate of vector map service compared with 1-node cluster.

During the map service performance test, different cluster environments and different concurrent numbers were designed to compare the response times. For the tile map test, four types of clusters were built The number of nodes in each cluster was 1, 2, 4, and 8, the concurrent request number in each cluster varies from 200 to 1600. When accessing the geologic tile map for Wuhan, the response time varied with the number of concurrent requests and the number of cluster nodes, the test results are provided in Fig 18 and Fig 19. For the geologic vector map test, five types of cluster were built. The number of nodes in each cluster was 1, 2, 4, 6, 8, and the concurrent request number in each cluster varied from 50 to 200. When accessing the geologic vector map for Wuhan in a geospatial box from 114.125602E to 114.500707E and from 30.453932N to 30.708764N, the response time also varied with the number of concurrent requests and the number of the cluster nodes. The test results are provided in Fig 20 and Fig 21.

The response time is used to evaluate the cluster performance, and the response time improving rate is used to evaluate the variation of the performance compared with a 1-node cluster. As shown in the following expression, *RTIR* ^*n*^ indicates the following: compared with a 1-node cluster, the response time improving rate of *n-node* cluster. ${RT}_{c}^{n}$ represents the response time of an *n-node* cluster when the concurrent number is *c*.

$$RTIR^{n}=(\frac{RT_{c}^{1}}{RT_{c}^{n}}-1)\times 100\%$$ (7)

From Fig 18 and Fig 20, it can be observed that the response time for the map service linearly increases with increasing concurrent number. The vector map service response time is substantially longer than the tile map response time. This is because the vector map service must perform spatial processing on the cluster server, the service must dynamically compute the map extent and clip map. In addition, the communication between nodes also increases with increasing number of cluster nodes, which could consume system resources and increase the response time. Fig 19 and Fig 21 show the performance improving rate compared with the 1-node cluster, these two figures demonstrate that the performance is clearly improved with an increasing number of cluster nodes, especially for the geologic tile map service.

#### Spatial Data Processing Performance

The polygon clip work flow was used to test the performance of spatial data processing in GSICCP. Polygon clip is a common operation in GIS processing; thus, utilizing the polygon clip to test the performance could closely represent the actual use situation. The entire environment was deployed as follows: 1) Hardware environment: includes a cluster manager server, task loader server and task executing server, where each server has 8 GB RAM and a 2.4 GHz CPU with 8 computing cores. 2) Software environment: the.Net and related geospatial middleware were deployed on the task-executing server machine, load Runner 11 (a task runner manager) was deployed on the task loader server, and the IGServer cluster manager was deployed on the cluster manager server.

During the performance test, the single work flow and batch work flow were tested to verify whether the polygon clip performance would vary with the number of cluster nodes. The geologic vector map for Luzhou, China, was adopted as the testing data. In this geologic vector map, the point layers include 185,159 features, the line layers include 644,802 features, and the polygon layers include 207,498 features. For the single work flow, each user sends a URL request, a free load node will process the request, and each request will execute the polygon clip operation only once. Three types of clusters were designed when executing the single workflow, the number of nodes in each cluster was 1, 2, and 3, and the concurrent number varied from 12 to 41. The test results are given in Table 2. For the batch work flow, each user sends a URL request, and different nodes in the cluster will execute the polygon clip operation in a distributed manner. In addition, the request will be executed more than once. Three types of cluster were designed when executing the batch workflow, the number of nodes in each cluster was 1, 2, and 3, and the concurrent number varied from 3 to 12. The test results are listed in Table 3.

**Table 2:** Performance test of different clusters for polygon clip operation in single workflow.

NN^a^	1			2			3		
Indicator	CN^b^	RT^c^	TP^d^	CN	RT	TP	CN	RT	TP
**Point**	12	58.407	163.269	29	57.569	329.615	41	58.368	495.577
**Line**	12	58.356	163	29	58.634	326.965	41	57.429	491.433
**Polygon**	12	60.333	157.508	29	58.305	321.139	41	58.724	481.958

^a^Node Number

^b^Concurrent Number

^c^Response Time (s)

^d^Throughput (bytes/s)

**Table 3:** Performance test of different clusters for polygon clip operation in batch workflow.

NN^a^	1			2			3		
e	CN^b^	RT^c^	TP^d^	CN	RT	TP	CN	RT	TP
**Point**	4	59.83	145.031	6	58.942	220.975	12	58.084	454.284
**Line**	4	59.607	148.891	6	59.724	219.596	12	59.261	445.97
**Polygon**	4	60.129	146.188	6	60.171	210.036	12	60.271	430.715

^a^Node Number

^b^Concurrent Number

^c^Response Time (s)

^d^Throughput (bytes/s)

Table 2 and Table 3 show the variation of the response time and throughput as a function of the cluster node and concurrent number in the polygon clip work flow. Overall, for a given time, a cluster with more nodes could process more user requests; in addition, the data throughput increased. During the performance test, the CPU rate was always lower than 75%, which means more cluster nodes could help improve the performance of the entire system.

### Discussion

Many geoprocessing services were built upon the architecture of GSICCP. The geological data service and geological processing service were introduced above. There are other services that have been deployed on the GSICCP such as a catalog service, geological production scheduling service, and GIS interoperation service. All these services and applications have greatly benefited from the geological work. Below, several intentions of this research are discussed.

It is evident that the newly emerged area of cloud computing can be implemented as easy-to-use tools[30]. Our research also adopts the concept of cloud computing to produce initial work; however, our work concentrates more on “how to build a geological cloud environment”, not “how to utilize an existing cloud environment well”. For historical reasons, substantial geological data and numerous services and applications have been developed and can be very complex to integrate. For a particular application or geological thematic problem, we can deploy such services and applications on existing cloud environments such as Azure[1,16,31]; however, for the entire geological domain, an open framework or platform that can integrate all geology-related data, services, and applications may be greatly beneficial. In such an environment, regardless of who integrates their service into the platform, some standards must be observed. This means that all integrated services use some common interfaces to share their information and obtain new information from other systems. Our research focuses on this objective, from hardware to the application. Considering data description, discovery, integration, management and sharing, according to the p2p node manager, we built a geological cloud environment. The entire cloud environment was deployed on more than 20 nodes. On the GSICCP, existing applications, such as geospatial data services, catalog services, geological production scheduling services, and GIS interoperation services, could achieve proper data sharing and cooperation. Furthermore, more geological systems could be integrated or developed within the GSICCP in the future to gradually form a geological service environment.

## Conclusions and Outlook

This study built a Geological Survey Information Cloud-computing Platform that integrates and shares distributed geological spatial data and services and provides users with related geological cloud services on software, platform and infrastructure levels, which are known as SaaS, PaaS and IaaS, respectively. Our work primarily focused on the following aspects: 1) the design of the Geological Survey Information Cloud-computing Platform structure, 2) the introduction of geological ontology theory to universally organize and describe the geological spatial data and to utilize the MapGIS platform to manage the geological spatial data, and 3) the design of the P2P node manager used to organize the computing and storage nodes in the GSICCP.

Using the developed GSICCP, related studies can be performed in the future to improve the performance of the entire system. The GSICCP integrated massive geological spatial data. Thus, a framework for large geological data mining has been established. In the future, a data mining application should be developed on the GSICCP to help mine geological knowledge and to expand geology-related information, knowledge, services and applications.
